# Misbalance of thyroid hormones after two weeks of exposure to artificial light at night in Eurasian perch *Perca fluviatilis*

**DOI:** 10.1093/conphys/coaa124

**Published:** 2021-01-07

**Authors:** Franziska Kupprat, Werner Kloas, Angela Krüger, Claudia Schmalsch, Franz Hölker

**Affiliations:** 1 Leibniz-Institute of Freshwater Ecology and Inland Fisheries, Müggelseedamm 310, 12587 Berlin, Germany; 2 Faculty of Life Sciences, Humboldt University, Invalidenstr. 42, 10099 Berlin, Germany

**Keywords:** Endocrine disruption, fish, freshwater, light pollution, thyroxine, triiodothyronine

## Abstract

Artificial light at night (ALAN) can affect the physiology and behavior of animals because it alters the natural rhythm of light and darkness. Thyroid hormones (TH) are partially regulated by the light information of photoperiod and are involved in metabolic adjustments to daily and seasonal changes in the environment, such as larval and juvenile development, somatic growth and reproduction. ALAN can change photoperiodic information and might thereby lead to changes in thyroid metabolism, but so far research on this topic is scarce. Therefore, we tested in two different experiments the effects of nocturnal illumination at a wide range of light intensities on TH in plasma of Eurasian perch (*Perca fluviatilis*). Total 3,3′,5-triiodo-L-thyronine (T3) was significantly affected by ALAN and reduced at the highest tested intensity of 100 lx after only two weeks of exposure. Although total L-thyroxine (T4) was not significantly affected, the ratio of T3 to T4 tended to slightly decrease at 100 lx. In a second low-light experiment ALAN did not have clear effects on T3, T4 or the ratio of T3 to T4 at intensities between 0.01 lx and 1 lx. The results show first signs of endocrine disruption in thyroid metabolism after a relatively short ALAN exposure of two weeks under high-intensity streetlight conditions. Misbalanced thyroidal status can have serious implications for metabolic rates as well as developmental and reproductive processes.

## Teaser

Artificial light at night can impair the physiology of animals. For the first time, we show that nocturnal illumination affects thyroid hormones as we found a reduction of triiodothyronine (T3) in Eurasian perch. Overall, the results indicate endocrine disruption by light pollution in freshwater fish.

## Introduction

Artificial light at night (ALAN) is an unprecedentedly increasing disturbance of natural nocturnal darkness (Gaston *et al.*, 2015, Hölker *et al.*, 2010). It mostly derives from centers of human activities, which are typically located in the vicinity of freshwater systems (Kummu *et al.*, 2011). Therefore, freshwater fish frequently experience nocturnal illumination, which can disturb the precise timing and optimization of their biological rhythms, for example daily and seasonal rhythms of hormones, such as melatonin (Brüning *et al.*, 2018a, Brüning *et al.*, 2015, Grubisic *et al.*, 2019, Kupprat *et al.*, 2020) or reproductive hormones (Brüning *et al.*, 2016, Brüning *et al.*, 2018b), but also food consumption or migratory behavior (Bergman, 1988, Riley *et al.*, 2012, Riley *et al.*, 2013). Light intensities of ALAN on the water surface of urban and suburban lakes or rivers normally range from 0.007 to 0.55 lx (indirect illumination by skyglow) up to 10 to 100 lx (direct exposure to a strong light source) (Hänel *et al.*, 2018, Hölker *et al.*, 2018). Hence, fish populations in such areas likely experience ALAN intensities <1 lx close to the water surface and can occasionally be exposed to even higher intensities ranging from 1 lx up to 100 lx (e.g. swimming directly beneath a strong light source). Exposure of fish depends on turbidity and swimming depth but specific exposure scenarios have not been quantified, yet. For comparison, daylight reaches a maximum of about 120 000 lx and ca. 800 lx at sunset (Brown, 1952, Gaston *et al.*, 2014, Grubisic *et al.*, 2019).

There are several reports of circadian or ultradian rhythms of thyroid hormones (TH) in blood plasma of teleost fish, for example in rainbow trout (*Oncorhynchus mykiss*) (Boujard and Leatherland, 1992, Cook and Eales, 1987, Gomez *et al.*, 1997, Laidley and Leatherland, 1988, Osborn *et al.*, 1978, Reddy and Leatherland, 2003), red drum (*Sciaenops ocellatus*) (Leiner and MacKenzie, 2001) or zebrafish (*Danio rerio*) (Jung *et al.*, 2016) and some studies report lunar rhythms of TH in smolting coho salmon (*Oncorhynchus kisutch*) (Farbridge and Leatherland, 1987, Grau *et al.*, 1981). Plasma TH further exhibit seasonal rhythms, which are positively correlated to sexual steroids during the annual reproductive cycle in many seasonally spawning teleosts (Cyr and Eales, 1996). Furthermore, seasonal profiles of plasma TH are available for a number of other teleost species (Bau and Parent, 2000, Dickhoff *et al.*, 1982, Eales and Fletcher, 1982, Osborn and Simpson, 1978, Özeren *et al.*, 2019), but no general pattern can be drawn from the available data. In Eurasian perch (*Perca fluviatilis*), for example, seasonal profiles of plasma TH revealed highest concentrations in summer and low levels during winter and spring and pikeperch (*Sander lucioperca*) had highest concentrations in late spring and fall and lowest concentrations in summer (Bau and Parent, 2000). Although a clear mechanistic understanding is still needed, light perception of photoperiod or moon phases has been proposed as one of the regulatory mechanisms for daily, lunar and seasonal TH rhythms (Grau, 1988, Laidley and Leatherland, 1988, Leatherland, 1994). Therefore, ALAN is likely to disturb these rhythms. Melatonin, which has a well-described direct neuroendocrine response to light and ALAN in teleosts (Grubisic *et al.*, 2019), might be an important regulatory component for rhythms of thyroid hormones; however, we know very little on this relationship for fish. In rodents, melatonin reduces thyroid function (Baschieri *et al.*, 1963, Vriend *et al.*, 1979, Wittkowski *et al.*, 1988) similar to amphibians in which melatonin is considered to be an antagonist of thyroid function (Wright *et al.* 2002). For rodents and birds, alterations in thyroid metabolism during long-day photoperiods, associated to low melatonin production, have been suggested as long-term timers in reproductive processes (Dardente *et al.*, 2014, Ouyang *et al.*, 2018, Wood and Loudon, 2014, Yoshimura, 2010). For example, in Siberian hamsters (*Phodopus sungorus*) short days reduced the gene expression of thyroid-stimulating hormone (TSH) receptor, which is associated with reduced gonadal development. Dim light at night (5 lx), however, increased expression of TSH receptors in short-day scenarios along with increased expression of reproductive hormones (Ikeno *et al.*, 2014).

In fish, TH are mainly known for regulation of developmental (differentiation and growth) and reproductive processes (Blanton and Specker, 2007, Campinho, 2019, Carr and Patiño, 2011, Power *et al.*, 2001). The role of TH in fish is best studied in the smoltification of Atlantic salmon (*Salmo salar*) and in the metamorphosis of flatfishes, in which thyroid disruption can inhibit or delay the onset and rate of metamorphosis and lead to serious malformations, for example in olive flounder (*Paralichthys olivaceus*) (Miwa and Inui, 1987). Research on TH metabolism under ALAN exposure is hence urgently needed to estimate the potential threat that animals in light polluted areas might be exposed to.

Structurally, TH are iodothyronines of which the biologically active forms are 3,3′,5-triiodo-L-thyronine (T3) and L-thyroxine (T4), the latter one being less biologically active. TH induce specific response mechanisms in various organs by binding to thyroid receptors and directly regulating gene expression in target cells. Thereby, especially T3 homeostasis in target cells is important for normal metabolic function (Brown *et al.*, 2004). However, T3 is not only directly produced by thyroid follicles because they mainly synthesize and secrete T4 rather than T3 upon stimulation by TSH. TSH is produced by the hypophyseal *pars distalis* representing the classical endocrine regulated hypothalamus-pituitary-thyroid-axis (Kloas *et al.*, 2009) but also by the photoreceptive *saccus vasculosus* in the hypothalamus of fish (Nakane *et al.*, 2013, Nakane and Yoshimura, 2014, O'Brien *et al.*, 2012). T4 is mostly catalyzed to T3 by deiodination in the outer ring of the T4 molecule and is often considered as a prohormone for T3.

Deiodination can be catalyzed by different isoforms of iodothyronine deiodinases (DIO). Type 1 deiodinase (DIO1) is thought to be rather unspecific as it catalyzes both outer- (ORD) and inner ring deiodination (IRD) with low activity levels (Orozco and Valverde-R, 2005). Type 2 deiodinase (DIO2) mainly catalyzes ORD and is thereby an ‘activating enzyme’ as it catalyzes ORD from the biologically less active T4 into the more active T3. Type 3 deiodinase (DIO3) instead is an inactivating enzyme by catalyzing IRD from the less active T4 to the inactive 3,3′,5′-trioiodo-L-thyronine (reverse T3) or from the active T3 to the inactive 3,3′-diiodothyronine (T2).

Overall, thyroid metabolism is likely to be regulated directly by endocrine as well as by daily and seasonal light information and such photic regulation might also be linked indirectly to melatonin. Exposure to ALAN can lead to modifications of both, light information and melatonin levels, but little is known about the impacts of ALAN on TH of fish. Therefore, we ran two different experiments in which *P. fluviatilis* were exposed to a wide range of ALAN intensities. In the first experiment fish were exposed to higher intensities of 1 lx, 10 lx and 100 lx and in the second one to lower intensities of 0.01 lx, 0.1 lx and 1 lx. Total T3 and total T4 were measured in plasma after two weeks in both experiments.

## Material and Methods

### Ethical statement

The care and use of experimental animals complied with German animal welfare laws, guidelines and policies as approved by the Berlin State Office of Health and Social Affairs (LAGeSo reference number G0055/16).

### Experimental fish

Eurasian perch (*P. fluviatilis*) from Lake Müggelsee (Berlin, Germany) were kept in 600-L indoor tanks with natural photoperiod (sunlight through windows and dark nights) prior to acclimation in the experimental setup (pre-acclimation, see below for details). According to the ‘new world atlas of artificial night sky brightness’ the surface of Lake Müggelsee experiences an illumination of ca. 0.003 lx in moonless clear nights (Falchi *et al.*, 2016), which lies in the lower range of suburban skyglow (Hänel *et al.*, 2018).

### Experimental setup

Individuals of *P. fluviatilis* (pubertal to young adults) were exposed to ALAN treatments in 80-L aquaria (length, 80 cm; width, 35 cm; height, 40 cm), which were covered with black foil to ensure independent light treatments. The lids of all aquaria were equipped with three fluorescent tubes to realize daylight intensities that reached up to 7000 lx at the water surface and were around 2900 lx averaged over 25 equally distributed points on the water surface. An additional fluorescent tube was installed for night-time illumination. Control levels were below detection limit of our luxmeter (ILT1700, Peabody, MA, USA), i.e. <0.00167 lx and are referred to as ‘0 lx’ in the following. Photoperiod was controlled by an automatic time switch system (Hager, Blieskastel, Germany). The experimental setup has been described before by Franke *et al.* (2013), providing quantitative and qualitative comparison between the experimental and natural conditions as well as details on the spectral composition of the light source (Biolux fluorescent tubes, Osram, Germany), which can be used to convert lux values into other illumination units (1 lx ≈ 3.7 mW m^−2^). It covers a large part of the spectral sensitivity of *P. fluviatilis* although their spectral sensitivity, compared to humans, is slightly more red-shifted (Cameron, 1982). Light intensity was adjusted by partial cover of the light source or using neutral density filter foil (Lee Colour Filter 299 1.2 N.D.) to maintain the spectral composition of the light. Since these methods do not change the spectral composition of the light source, lux can be used as the unit of illuminance for comparison across different light intensities.

#### (1) Exposure to high intensities of ALAN (‘high ALAN experiment’)

Fertilized egg ribbons of *P. fluviatilis* were collected from Lake Müggelsee (Berlin, Germany) in March 2015 to raise parasite-free fish as described by Vivas Muñoz *et al.* (2019). Pre-acclimation in 600-L indoor tanks lasted two weeks. The experiment was run twice in December 2016 and in January 2017, with each treatment in duplicate during the first run and in triplicate during the second run (i.e. *n* = 5 for each treatment). The experimental setup consisted of 12 identical 80-L aquaria with a tap water flow-through of ca. 10 L h^−1^ and water temperature of ca. 16 °C. Each aquarium was stocked with six fish which were allowed to acclimate for two weeks without illumination during the night (0 lx) followed by two weeks of experimental conditions with the respective nocturnal light intensity or controls without illumination according to Brüning *et al.* (2015). The fish weighed 69.0 ± 18.4 g with a standard length of 15.3 ± 1.3 cm (mean ± standard deviation, *n* = 120). Fish were fed with commercially available food (Aller Silver, 3 mm, Emsland-Aller, Golßen, Germany) twice a day at a rate of 0.5% of their body mass until feeding stopped 24 h prior to sampling to minimize effects of feeding. Full daylight was realized from 09:00 to 15:00 with a simulated dawn or dusk period by dimming over 3 h each starting at 06:00 or 15:00, respectively. This mimics December conditions of the natural photoperiod in Lake Müggelsee (52° 26′ N, 13° 39′ E). Nocturnal illumination of 1 lx, 10 lx or 100 lx at the water surface was from 18:00 till 06:00.

#### (2) Exposure to low intensities of ALAN (‘low ALAN experiment’)

*Perca fluviatilis* from Lake Müggelsee were caught by electro fishing and fish traps between July and September 2017 and fed twice a day with frozen blood worms. Pre-acclimation lasted 2 to 9 weeks. Thirty fish with an individual mass of 16.8 ± 4.1 g and standard length of 10.6 ± 0.9 cm (mean ± standard deviation, *n* = 720) were transferred to each 80-L aquarium. Fish were fed twice a day during acclimation (two weeks) and the water flow-through was adjusted to 10 L h^−1^. The temperature during acclimation and experimental exposure to ALAN was ca. 16 °C. Photoperiod was adjusted to October conditions with full daylight from 09:30 to 18:30 with a 3 h dawn or dusk period starting at 06:30 or 18:30, respectively. After acclimation, nocturnal illumination of 0.01 lx, 0.1 lx or 1 lx average intensity on the water surface was switched on from 21:30 until 06:30; controls were not illuminated during night (0 lx). During the two weeks of experimental illumination the water flow-through was reduced to 4 L h^−1^ in order to allow water-based melatonin measurements, which are described by Kupprat *et al.* (2020). To maintain good water quality, animals were not fed during the low flow-through in the two weeks of experimental illumination. The same experiment was performed twice—in October and November 2017—with each treatment in triplicates for both runs (i.e. *n* = 6 for each treatment).

### Sampling

Fish were randomly sampled on two consecutive mornings between 09:00 and 12:00 in the high ALAN experiment and in two consecutive nights between 22:00 and 04:00 in the low ALAN experiment. In the high ALAN experiment, all fish were sampled and blood sampling of one aquarium was completed within 15 min. In the low ALAN experiment, body mass and length were measured of all fish, blood was only taken from the first 15 fish and blood sampling of one aquarium was completed within 35 min. Five to six plasma samples from each aquarium, i.e. *n* = 35–36 for each experimental group, were analyzed. Males (m) and females (f) were distinguished by visual inspection of the gonads. In premature fish (nd) gonads could not be differentiated. In the low ALAN experiment sex was not determined for all fish and thus this information is not available for three blood samples (na). Blood (500–1000 μL) was taken from the caudal vein with heparinized syringes and transferred to a clean tube containing 1–2 mg Na_2_EDTA (EDTA). Blood and EDTA were mixed by shaking and centrifuged at 7500 x *g* for 5 min at 4 °C. The plasma was transferred to a new tube and immediately frozen in liquid nitrogen and then stored at −80 °C until analyzed.

### Extraction

Extraction was performed at room temperature. Blood plasma was thawed on ice and after vortexing, 70 μL of plasma were mixed with 17.5 μL protection solution containing dithiothreitol, ascorbic acid, citric acid, each at 25 g L^−1^, following previous protocols by Noyes *et al.* (2014) and Wang and Stapleton (2010). Then 140 μL of acetonitrile were added to the tube and vortexed for 1 min. 420 μL of ethyl acetate were added to the mixture followed by vortexing again for 1 min. After centrifugation at 4500 x *g* for 10 min, the upper phase was transferred to a new tube and the extracts were dried under vacuum at 45 °C (Concentrator plus, Eppendorf, Hamburg, Germany). The ethyl acetate extraction was repeated once in the first tube and the upper phase was added to the first extract and further dried. The dried extracts were resolved in 70 μL methanol and shaken for 1 h on a 3D shaker (Polymax 1040, Heidolph Instruments, Schwabach Germany). Samples were centrifuged for 1 min at 10000 x *g* and 60 μL were transferred to an insert (250 μL) in an HPLC vial and stored at −20 °C until further use.

### LC–MS/MS measurement

We measured total T3 and total T4 from extracted plasma in a triple quadrupole tandem mass spectrometer (LC–MS/MS; 1290 Infinity II UHPLC, 6470 Triple Quadrupole Jetstream, Agilent, Santa Clara, CA, USA) with a C18 separation column (ZORBAX Eclipse XDB C18, Agilent). The injection volume was 2 μL and the flow rate was 0.4 mL min^−1^. For LC separation, 0.1% formic acid was used as the aqueous mobile phase (A) and acetonitrile with 0.1% formic acid was used as the organic mobile phase (B). The gradient was realized as follows: 0 min 90% A, 4 min 40% A, 5 min 40% A, 7 min 90% A, 8 min 90% A, 9 min 20% A, 12 min 20% A, 13 min 90% A, 15 min 90% A. MS/MS responses of target analytes were evaluated by electrospray ionization (ESI) in positive ion mode using multiple reaction monitoring (MRM) transitions. The transitions of T3 were at 651.8–605.8 or 651.8–353.0 with collision energy of 19 eV or 40 eV for quantifier ions or qualifier ions, respectively, and fragmentor voltage of 158 V. The T4 transitions were at 777.7–731.7 or 777.7–633.7 with collision energy of 23 eV or 35 eV, respectively and fragmentor voltage of 165 V. T3 was quantified at the lower and T4 at the higher transition.

A calibration curve between 0.5 and 25 ng mL^−1^ was prepared in methanol. The limit of quantification (LOQ)was 0.5 ng mL^−1^ for both, T3 and T4. However, after measuring the high ALAN samples there was a power outage and afterwards for most of the low ALAN samples the LOQ of T4 was 1 ng mL^−1^.

### Validation of methodology

Prior to measuring the samples we did a small spike and recovery experiment of three randomly chosen plasma samples (one high ALAN and two low ALAN samples) and three pools of plasma from *P. fluviatilis* of the same stock as the experimental animals of the low ALAN experiment. Subsets of each of the three plasma pools were spiked before extraction with either a high (20 ng mL^−1^) or low (5 ng mL^−1^) end concentration of synthetic T3 or T4 in the spiked plasma samples. Recovery of post-extraction spikes is expressed as the percentage of the T3 or T4 from the non-spiked plasma samples subtracted from the T3 or T4 in the spiked plasma sample relative to the theoretical amount of spiked T3 or T4. Extraction efficiency was calculated likewise from pre-extraction spikes. Extraction efficiency determined by pre-extraction spikes was 68.0 ± 5.1% for T3 and 68.2 ± 3.0% for T4. Recovery from post-extraction spikes was increased by 5.6 ± 4.1% for T3 and 22.3 ± 7.0% for T4. Variation in recovery in post-extraction spikes was larger in T4 than in T3. We added antioxidants to the extraction solution, but enzymatic and non-enzymatic conversion from T4 to T3 cannot be fully excluded.

Solvents were obtained in ultrapure LC–MS grade from Carl Roth GmbH (Karlsruhe, Germany). All other chemicals were mainly obtained from Sigma-Aldrich Chemie GmbH (Taufkirchen, Germany).

### Data handling

Measurements were evaluated with Agilent MassHunter Workstation software (Version B.09.00).

In the high ALAN experiment, two T4 measurements were below the LOQ of 0.5 ng mL^−1^. In the low ALAN experiment, two samples were below 0.5 ng mL^−1^ T3. Those measurements were excluded from the data analysis. For T4, 27% of the low ALAN samples were below the LOQ and as these missing values were homogenously distributed over all treatment groups (0 lx, 25.7%; 0.01 lx: 30.6%; 0.1 lx: 22.2%; 1 lx: 34.3%), we decided to analyze available cases only. In the respective graph (Figure 2, middle panel) a dotted line depicts an alternative median, which takes into account the samples below LOQ. For this, all non-quantified samples were attributed a fixed constant of the respective LOQ (0.5 or 1 ng mL^−1^) divided by two.

### Statistical analysis

Data were log-transformed to meet the assumptions of statistical testing, i.e. normal distribution and homogeneity of residual variance. Each parameter (log(T3), log(T4) and log(ratio T3/T4) for high and low ALAN, respectively) was individually modeled in a linear mixed model (LMM) with treatment, body mass and sex as fixed effects and ‘aquarium nested in run’ as random effects. Models were selected by dropping one factor and comparing the two models. Models were selected according to lowest AIC and based on the LLRs and p-values of the analysis of variance (ANOVA) comparing two models (significance at p < 0.05) (Zuur *et al.*, 2009). If addition of treatment and body mass or sex did not explain significantly less variance than an interaction, additive fixed effects were modeled. If removal of body mass or sex did not significantly worsen the model, the factor was not included in the model. Treatment was kept as fixed effect in every model as it was the hypothesized effect we aimed to test for. Random effects were also kept in all LMMs to account for the data structure. In case of significant treatment, body mass or sex effects, post-hoc tests using Tukey’s correction compared every treatment or sex to every other within one experiment (Lenth, 2019). Treatment, sex, body mass and random effects as well as marginal and conditional R^2^ values (Barton, 2018) for each LMM are specified in Table 1 (page 18). Full model specifications for each parameter as well as full results of the post-hoc tests are given in the supplementary material.

**Table 1:** Treatment effects, sex effects, body mass effects and random effects and the marginal and conditional R^2^-values for linear mixed models of log-transformed total triiodothyronine (log(T3)), total thyroxine (log(T4)) and the ratio of T3/T4 (log(T3/T4) in blood plasma of *P. fluviatilis* exposed to different intensities of ALAN in two different experiments (‘high ALAN experiment’ or ‘low ALAN experiment’). An asterisk marks an interaction of two fixed factors

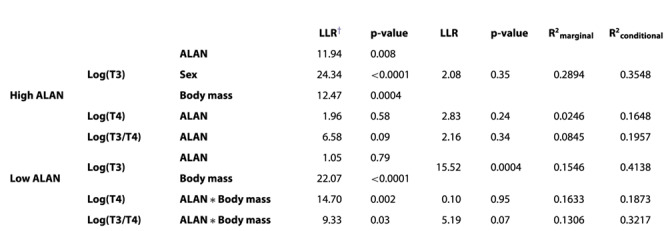

^†^Log-likelihood ratio

### Note on the differences between the two experiments

Since several experimental variables (size, life history, feeding regimes, sampling time of day, seasonal timing of experiments, water flow-through) differed across the high and the low ALAN experiment, it is important to compare treatments only with the respective control and avoid comparisons across experiments. Originally, we planned the high ALAN experiment with a wild population from Lake Müggelsee like in the low ALAN experiment and in earlier studies (Brüning *et al.*, 2016, Brüning *et al.*, 2015, Brüning *et al.*, 2018b). Yet, in fall 2016 we did not catch enough wild *P. fluviatilis* for the experiment and worked with lab-raised fish instead, which were pre-conditioned to dry feed. In contrast, the wild *P. fluviatilis* were fed with frozen blood worms because they could not be conditioned to dry feed. Experimental fish were not fed during the low ALAN experiment to maintain water quality under the low water flow-through which was necessary to measure melatonin from the tank water (Kupprat *et al.*, 2020). It was not necessary to reduce the water flow-through and starve the experimental fish in the high ALAN experiment because changes of nocturnal melatonin were known from a previous study (Brüning *et al.*, 2015). In the high ALAN experiment, samples were taken in the mornings after fish were exposed to ALAN for the entire night. However, as there was only one effect at 100 lx in the high ALAN experiment, samples were taken at night at direct application of nocturnal illumination in the low ALAN experiment.

## Results

### ALAN effects

**Figure 1:**
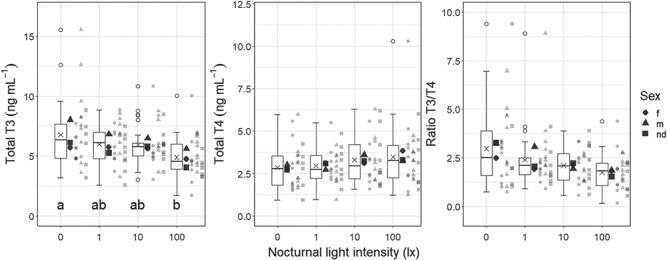
**Total triiodothyronine (T3) and thyroxine (T4) in blood plasma of *P. fluviatilis* under different light pollution scenarios (‘high ALAN experiment’).** Samples were all taken throughout the morning (09:00–12:00). Boxplots display data for each treatment and ‘X’s inside the boxes indicate the mean (T3: *n* = 26 for 0 lx and 10 lx, *n* = 27 for 1 lx and 100 lx; T4 and ratio T3/T4: *n* = 26 for all treatments). Boxplots are limited by the 25% and 75% quartile, with a horizontal line as the median and whiskers depicting the 1.5x interquartile ranges (IQR); outliers > 1.5x IQR are indicated by circles. Different shapes of big points indicate the mean of each sex (m—males, f—females, nd—not differentiated (premature fish)), whereas small points represent individuals (T3 (T4 and ratio T3/T4): *n* = 34 (34) for f, *n* = 40 (39) for m, *n* = 32 (31) for nd). Different letters indicate significant differences between treatments (Tukey’s post-hoc, p < 0.05).

In the high ALAN experiment, mean T3 was significantly lowered by 28% at 100 lx illumination as compared to the dark control treatment (LMM ALAN effect: LLR = 11.94, p = 0.008; Tukey’s post-hoc: 0 lx vs. 100 lx: p = 0.01, Figure 1). Differences of 1 lx or 10 lx compared to 0 lx were not significant (Tukey’s post-hoc: 0 lx vs. 1 lx: p = 0.69, 0 lx vs. 10 lx: p = 0.80), neither were differences between 100 lx and 1 lx or 10 lx (Tukey’s post-hoc: 1 lx vs. 100 lx: p = 0.08, 10 lx vs. 100 lx: p = 0.06). Mean concentrations of T4 slightly increased by 21% at 100 lx and the mean ratio of T3/T4 decreased by 41% at 100 lx compared to controls without ALAN, although both without statistically significant treatment effects (LMM ALAN effects: T4—LLR = 1.96, p = 0.58; T3/T4—LLR = 6.38, p = 0.09, Table 1).

**Figure 2:**
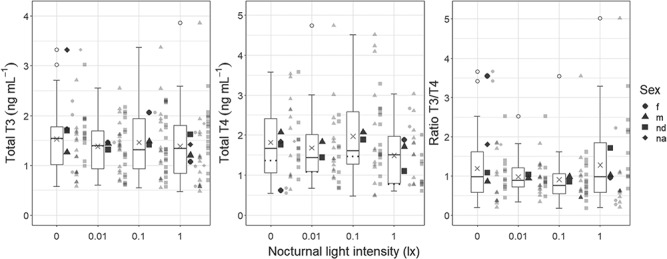
**Total triiodothyronine (T3) and thyroxine (T4) in blood plasma of *P. fluviatilis* under different light pollution scenarios (‘low ALAN experiment’).** Samples were all taken throughout the night (22:00–04:00). Boxplots display data for each treatment and ‘X’s inside the boxes indicate the mean (T3: *n* = 34 for 0 lx, *n* = 36 for 0.01 lx and 0.1 lx, *n* = 35 for 1 lx; T4 and ratio T3/T4: *n* = 26 for 0 lx and 0.1 lx, *n* = 25 for 0.01 lx, *n* = 23 for 1 lx). Boxplots are limited by the 25% and 75% quartile, with a horizontal line as the median and whiskers depicting the 1.5x interquartile ranges (IQR); outliers > 1.5x IQR are indicated by circles. Different shapes of big points indicate the mean of each sex (m—males, f—females, nd—not differentiated (premature fish), na—not available (sex not determined)), whereas small points represent individuals (T3 (T4 and ratio T3/T4): *n* = 8 (5) for f, *n* = 66 (52) for m, *n* = 63 (44) for nd, *n* = 3 (1) for na). There were no significant differences across treatments in any parameter. Dotted horizontal lines in the middle panel (total T4) display medians which take into account measurements below the limit of quantification (*n* = 35 for 0 lx and 1 lx, *n* = 36 for 0.01 lx and 0.1 lx).

In the low ALAN experiment, no ALAN effects were detected for T3 (LMM ALAN effects: LLR = 1.05, p = 0.79, Table 1, Figure 2). An interaction of ALAN and body mass significantly explained variation of T4 and T3/T4 (LMM ALAN*Body mass effects: T4—LLR = 14.70, p = 0.002; T3/T4—LLR = 9.333, p = 0.03). T4 decreased with increasing body mass at 0 lx and 1 lx, but increased with increasing body mass at 0.01 lx and 0.1 lx. Overall, T3/T4 overall increased with increasing body mass, with the weakest increase at 0.1 lx and the steepest increase at 1 lx and a slight decrease at 0.01 lx (for details, see supplementary material).

### Body mass effects

In the high ALAN experiment, body mass significantly explained variance of T3 (LMM body mass effect: LLR = 12.47, p = 0.0004) but the slope was very flat. Body mass was not kept as fixed effect in the model selection of the LMMs of T4 or T3/T4 in the high ALAN experiment as it did not significantly explain more variance.

In the low ALAN experiment, body mass also significantly explained variance of T3 (LMM body mass effect: LLR = 22.07, p < 0.0001) and T4 and T3/T4 had the above described interaction with ALAN effects.

### Sex effects

In the high ALAN experiment, the sex ratios of the experimental fish for which TH were determined were 32%:38%:30% (females (f):males (m):not differentiated (nd)) and in the low ALAN experiment there were only few females resulting in sex ratios of 6%:47%:45%:2% (f:m:nd:na (not available)) for T3 or 5%:51%:43%:1% for T4, respectively (detailed number for each sex listed per treatment in the supplementary material). Interactions between sex and ALAN did not improve the models and were, thus, not included to keep the model as simple as possible. Sex effects were only significantly improving the model for T3 of the high ALAN experiment (LMM sex effect: LLR = 24.34, p < 0.0001) where males had significantly higher levels of T3 than females or not differentiated fish (Tukey’s post-hoc: f vs. m: p = 0.005; nd vs. m: p = 0.006).

### Random effects

Random effects did not significantly explain TH variance in the high ALAN experiment or variances of T4 or T3/T4 in the low ALAN experiment. For T3 in the low ALAN experiment, random effects significantly explained variance (LMM random effects: LLR = 15.52, p = 0.0004). Ca. 31% of the unexplained variance (the variance that was not explained by the fixed effects) was explained by variance across the two runs (October and November); repeatability for aquarium effects was negligibly low.

## Discussion

Exposure to a high intensity of nocturnal illumination (100 lx) caused a significant reduction of T3 in the blood plasma of *P. fluviatilis* as compared to controls without illumination after two weeks. For T4 there was no significant effect of the light treatments, but a trend for a decrease of the T3/T4 ratio could be observed. In a second experiment with lower intensities of ALAN there were no clear effects on TH.

### ALAN effect

The significant decrease in T3 at 100 lx with stable T4 levels could be explained by differences in rates of TH synthesis, secretion, conversion and excretion. One mechanistic explanation could be a reduced extrathyroidal conversion of T4 into T3 by ORD. The DIO2/DIO3 ratio could be affected or just one of the deiodinases. The trend of decreasing T3/T4-ratio further implicates alterations in deiodination activities. In contrast, in rodents short photoperiods and melatonin rather down-regulate *Dio2* expression and long-photoperiods lead to high expression of *Dio2* (Dardente *et al.*, 2014). Additionally, long photoperiods correlate with increased levels of TSH and T3 in birds and mammals (Nakao *et al.*, 2008, Yoshimura, 2010). Accordingly, DIO2 activity and T3 should increase under ALAN (Ouyang *et al.*, 2018), but our results rather suggest the opposite, indicating mechanistic differences between mammals or birds and teleost fish in the light-dependent regulation of thyroid metabolism. Overall, a TH-antagonistic role of melatonin, like it is described for rodents and amphibians, cannot be confirmed for *P. fluviatilis* as melatonin was reduced by ALAN (Brüning *et al.*, 2015, Kupprat *et al.*, 2020) and TH were reduced or unchanged and did not increase. The observed changes in plasma TH are probably not a result of changed thyroid stimulation by TSH because this would likely result in an overall decrease or increase in both, T3 and T4. In future experiments, a full profile of thyroid endpoints, including plasma and peripheral tissue levels of all TH and activities of deiodinases as well as gene expression of *tsh* or deiodinases, may help to identify processes leading to the observed change.

It is interesting that total T3 is affected by ALAN already after two weeks, but the effect was significant only at 100 lx, which is at the upper end of realistic light pollution scenarios (Grubisic *et al.*, 2019, Hänel *et al.*, 2018) and *P. fluviatilis* will most likely only occasionally experience these intensities. It is in principle possible that ALAN effects are accumulating over time, i.e. an effect like the one at 100 lx could be detected at lower and more typical ALAN intensities after longer exposure (e.g. several months). Moreover, shorter exposure times to ALAN of only a few hours or days could be interesting to test for a time-specific dose–response relationship.

In the low ALAN experiment, ALAN effects were interacting with the effect of body mass on T4 and T3/T4, but there was no clear pattern along the gradient of ALAN intensities. Thus, the current data do not seem to allow a clear interpretation of the relationship between TH, body mass and ALAN. However, our data indicate that consideration of body mass can improve TH analyses.

Seasonal effects of ALAN on TH may be considered in future studies to identify if TH dynamics of *P. fluviatilis* can be also characterized by a photo-labile period, where fish are particularly susceptible to additional light at night (Falcón*et al.*, 2010), as suggested for suppression of sexual hormones in *P. fluviatilis* (Brüning *et al.*, 2016, Brüning *et al.*, 2018b). In *S. salar*, experiments on the timing of photoperiod manipulation of TH have identified a photo-labile period (McCormick *et al.*, 1987). Thus, ALAN may have particularly strong effects on TH of *P. fluviatilis* during a sensitive time window.

For a critical interpretation of our data, possible daily rhythms of plasma TH in *P. fluviatilis* need to be considered. Although to our knowledge no data on daily TH rhythms in *P. fluviatilis* is available, circadian rhythms were reported for *O. mykiss* (Osborn *et al.*, 1978) or *D. rerio* (Jung *et al.*, 2016), and also ultradian rhythms were reported for *O. mykiss* (Gomez *et al.*, 1997, Laidley and Leatherland, 1988). Potential phase shifts of such rhythms can affect experimental results (Leatherland, 1994), for example if absolute production of TH was not affected but only a shift in the production peaks occurred under ALAN exposure. In this context, it must be mentioned that the daily peaks of plasma T3 can occur at different times than the peaks of T4, e.g. reported in *O. mykiss* (Boujard and Leatherland, 1992, Cook and Eales, 1987, Reddy and Leatherland, 2003), and *S. ocellatus* (Leiner and MacKenzie, 2001). Furthermore, peaks of plasma T3 and T4 can depend on a combination of light information and feeding times (Boeuf and Le Bail, 1999, Cook and Eales, 1987) and TH levels were lowered in Arctic charr (*Salvelinus alpinus*) (Eales and Shostak, 1985) and *O. mykiss* (Cook and Eales, 1987) when fish were starved. Since fish were not fed in the low ALAN experiment (due to water-based measurements of melatonin (Kupprat *et al.*, 2020)), starvation may have kept TH levels low enough to diminish effects besides the low levels of ALAN and long sampling times. In comparison, circadian rhythmicity of melatonin was still measured under starvation conditions for *P. fluviatilis* with lowered amplitudes at 0.01 lx and 0.1 lx (without phase shifts) and at 1 lx first indications of a phase shift were reported (Kupprat *et al.*, 2020). At 10 lx and 100 lx, rhythms were completely depleted (Brüning *et al.*, 2015). A strong regulatory role of melatonin on TH production in *P. fluviatilis* seems unlikely, because if melatonin was a main regulator of TH production, ALAN effects on TH would have to be expected at all tested intensities, especially > 1 lx at which not only the absolute amount of melatonin was affected, but also its circadian pattern.

### Ecophysiological implications of misbalanced thyroid metabolism

The reduction of T3 at 100 lx can have critical effects on the metabolic rate, and developmental or reproductive processes of *P. fluviatilis*. Body mass was a significant fixed effect in the LMM analysis of T3, but there were no significant ALAN-related changes of body mass within the two weeks of the experiment. A reduction of T3 would result in reduced occupancy of thyroid receptors in target cells and this would eventually lead to a reduced gene expression of TH-regulated proteins. The identification and classification of these proteins remains subject to future research but if thyroid metabolism is impaired over longer times, developmental and growth processes may change drastically (Power *et al.*, 2001). ALAN-induced changes in thyroid metabolism could be particularly harmful for fish species that undergo a dramatic metamorphosis, in which TH play an important role, e.g. salmonids (Laudet, 2011, Lorgen *et al.*, 2015) or flatfishes (Inui and Miwa, 1985, Schreiber, 2006). In aquacultural production of *S. salar* and masu salmon (*Oncorhynchus masou*), photoperiod manipulations are used as a tool to induce smoltification, which is necessary to allow physiological adjustment for the transfer to seawater and optimize growth (Boeuf and Gaignon, 1989, McCormick and Saunders, 1990, McCormick *et al.*, 1987, Okumoto *et al.*, 1989). However, TH likely also regulate less spectacular larval-juvenile transformations and juvenile-adult developments of most other teleost species (Laudet, 2011). For instance, in *D. rerio*, hypothyroid conditions (comparable to reduced T3 at 100 lx in our experiments) in eggs caused altered pace of development, changes in pigmentation and malformations in lower jaws (Carr and Patiño, 2011, Mukhi and Patiño, 2007, Walpita *et al.*, 2009, Walpita *et al.*, 2007) and paired fin development in larvae is also TH-dependent (Brown, 1997). In amphibian metamorphosis, where TH are key in regulating tail shrinking, limb growing and changes in cranial structure, an impairment of the thyroid axis can have drastic consequences, for example in the rate of metamorphosis (Fort *et al.*, 2000, Kloas, 2002, Opitz *et al.*, 2005). In the American toad (*Anaxyrus americanus*), ALAN exposure of 3–15 lx reduced metamorphic duration and reduced post-metamorphic growth (Dananay and Benard, 2018), but a link to changes in TH (or melatonin) by ALAN has not been considered. Whether an ALAN-induced misbalance of TH would impair growth, larval-juvenile transformations, or reproductive processes in *P. fluviatilis* and other fish is an interesting objective for future research.

Changes in the thyroid cascade are often used as a sensitive biomarker of exposure to chemical pollutants (Brown *et al.*,2004). Comparable effects with lower T3 and stable or increasing T4 in teleost plasma were observed after exposure to low pH (Brown *et al.*, 1989, Brown *et al.*, 1984, Brown *et al.*, 1990), polychlorinated biphenyls (Adams *et al.*, 2000, Leatherland and Sonstegard, 1978), chemical effluents (Carletta *et al.*, 2002, Zhou *et al.*, 2000), kerosene (Peter *et al.*, 2007) as well as cortisol (Brown *et al.*, 1991) and estradiol (Cyr and Eales, 1990, Cyr *et al.*, 1988b, Leatherland, 1985). As these substances are known as endocrine disruptors, these similarities to our results underline the potential of ALAN to act as an endocrine disruptor. Currently, the public and political acknowledgement of light pollution as an environmental threat is limited and regulatory measures are, if at all, recommendations and not legally binding law (Kyba *et al.*, 2014, Schroer *et al.*, 2020). From the data presented here, TH in fish does not seem to be an exceptionally sensitive endpoint for light pollution, because an effect was only detectable at a relatively high level of illumination (100 lx). Pre-metamorphic amphibians might be more sensitive animal models to investigate effects of ALAN on TH (e.g. OECD, 2009). In fish, other hormonal endpoints appear to be more relevant with regard to ALAN, for example sex steroid blood concentration, gene expression of gonadotropins and especially nocturnal melatonin production, which are affected at low ALAN intensities within the range of typical skyglow illumination (< 2 lx) (Brüning *et al.*, 2016, Brüning *et al.*, 2018b, Grubisic *et al.*, 2019, Kupprat *et al.*, 2020). Thus, future regulatory light pollution measures need to consider the effects on the entire biodiversity in and along freshwater systems (Reid *et al.*, 2018) because light pollution has already been evidenced to affect other aquatic organisms such as microbes, algae, aquatic insects, amphibians and land-water interactions, with a potential for ecosystem-level changes through bottom-up and top-down processes (e.g. Grubisic *et al.*, 2017, Hölker*et al.*, 2015, Manfrin *et al.*, 2017, Touzot *et al.*, 2020).

### Ratio of T3/T4

Besides the decreasing trend of the T3/T4-ratio with increasing ALAN intensity, we aimed to put the ratio levels of the control treatments into context. Our experiments revealed similar or higher levels of T3 compared to T4 in the plasma of *P. fluviatilis* and the ratio of T3/T4 lies roughly at 2.5:1 in the high ALAN experiment and in the low ALAN experiment at 1:1, although the real ratio in the low ALAN experiment is likely to be higher as ca. 27% of T4 (but not T3) measurements were below the limit of quantification. Another study, which measured plasma T3 and T4 from *P. fluviatilis* throughout one year *in situ*, reported mean ratios of 1:1 in winter up to 1:12 in early summer (Bau and Parent, 2000). Since there is no reference value available for the ratio of T3/T4 in teleost fish, we have reviewed relevant literature to better place our results in perspective. Most studies on fish TH report T3 and T4 values with a T3/T4 ratio of about 1:1 to 1:5 (Adams *et al.*, 2000, Bau and Parent, 2000, Boujard and Leatherland, 1992, Cook and Eales, 1987, Cyr *et al.*, 1988a, Eales and Fletcher, 1982, Eales and Shostak, 1985, Farbridge and Leatherland, 1987, Gomez *et al.*, 1997, Hoseini *et al.*, 2016, Laidley and Leatherland, 1988, McCormick and Saunders, 1990, McCormick *et al.*, 1987, Osborn and Simpson, 1978), but also extreme ratios from 16:1 (Cyr *et al.*, 1988a) to 1:10 (Bau and Parent, 2000, Jung *et al.*, 2016, Zhao *et al.*, 2016) or up to 1:20 (Arkoosh *et al.*, 2017, Boujard and Leatherland, 1992, Chen *et al.*, 2017) have been published. Still, higher T3 than T4 levels are not unusual and have been reported frequently in teleost plasma (Bau and Parent, 2000, Cook and Eales, 1987, Cyr *et al.*, 1988a, Eales and Fletcher, 1982, Eales and Shostak, 1985, Leiner and MacKenzie, 2001, Osborn and Simpson, 1978, Osborn *et al.*, 1978, Reddy and Leatherland, 2003), as well as in fertilized eggs of teleost fish (Power *et al.*, 2001, Weber *et al.*, 1992). Overall, the ratio seems to depend on several factors, such as species, time of day or year, photoperiod, but also body mass and feeding regime. Hence, the high T3/T4 ratio in the high ALAN experiment is not unusual for teleost fish but is not in line with the measurements from an earlier study (Bau and Parent, 2000). The 1:1 ratio in the low ALAN experiment (in October conditions) matches the results of Bau and Parent (2000) und is also the most commonly reported T3/T4 ratio for teleost fish.

### Sex effects

Males had significantly higher levels of T3 than females and non-differentiated fish in the high ALAN experiment, but T4 was not significantly affected by sex. These results might be related to suppression of T3 (but not T4) by estradiol in females, as reported for *O. mykiss* (Cyr and Eales, 1990, Cyr and Eales, 1996, Cyr *et al.*, 1988b, Leatherland, 1985). In the low ALAN experiment, however, these sex effects were not confirmed, probably due to low number of females (5%).

## Conclusion

Our results are among the first steps towards understanding the impacts of ALAN on thyroid metabolism in fish. High intensities of ALAN at 100 lx led to an increasing misbalance of thyroid metabolism in *P. fluviatilis* after only two weeks with decreased plasma T3 and stable plasma T4. However, ALAN intensities of 10 lx and 1 lx did not show a significant decrease. In a second experiment with even lower intensities, representing realistic skyglow exposure, there were no clear effects of ALAN on TH. Still, it is possible that a longer exposure to lower intensities causes a similar reduction of T3 as measured after two weeks at a high intensity. If the misbalance of thyroid metabolism as measured at 100 lx persisted in the long run, metabolic mismatches could lead to impaired developmental (larval-juvenile development) and reproductive processes. Teleost species undergoing a distinctive metamorphosis, such as flatfishes or salmonids could be particularly vulnerable to thyroid-related ALAN effects.

## Supplementary Material

Kupprat_et_al_ConsPhys_Supplementary_material_re-revised_coaa124

## Notes

Author contributions: Conceptualization: F.H., F.K., W.K.; Funding acquisition: F.H., W.K.; Experiments: F.K.; Lab analyses: A.K., C.S., F.K.; Provision of material and equipment: A.K., F.H., W.K.; Data analysis: F.K.; Initial draft preparation: F.K.; Manuscript editing and reviewing: all authors.

## References

[ref1] Adams BA, Cyr DG, Eales JG (2000) Thyroid hormone deiodination in tissues of American plaice, *Hippoglossoides platessoides*: characterization and short-term responses to polychlorinated biphenyls (PCBs) 77 and 126. Comp Biochem Physiol C-Toxicol Pharmacol 127: 367–378. 10.1016/S0742-8413. 11246509

[ref2] Arkoosh MR, Van Gaest AL, Strickland SA, Hutchinson GP, Krupkin AB, Dietrich JP (2017) Alteration of thyroid hormone concentrations in juvenile chinook salmon (*Oncorhynchus tshawytscha*) exposed to polybrominated diphenyl ethers, BDE-47 and BDE-99. Chemosphere 171: 1–8. 10.1016/j.chemosphere.2016.12.035. 28006665

[ref3] Barton K (2018). MuMIn: multi-model inference. R package version 1.42.1.

[ref4] Baschieri L, de Luca F, Cramarossa L, de Martino C, Oliverio A, Negri M (1963) Modifications of thyroid activity by melatonin. Experientia 19: 15–17. 10.1007/bf02135330. 13969822

[ref5] Bau F, Parent J-P (2000) Seasonal variations of thyroid hormone levels in wild fish. Comptes Rendus de l'Académie des Sciences - Series III - Sciences de la Vie 323: 365–372. 10.1016/s0764-4469(00)00137-2. 10803347

[ref6] Bergman E (1988) Foraging abilities and niche breadths of two percids, *Perca fluviatilis* and *Gymnocephalus cernua*, under different environmental conditions. J Anim Ecol 57: 443–453. 10.2307/4916.

[ref7] Blanton ML, Specker JL (2007) The hypothalamic-pituitary-thyroid (HPT) axis in fish and its role in fish development and reproduction. Crit Rev Toxicol 37: 97–115. 10.1080/10408440601123529. 17364706

[ref8] Boeuf G, Gaignon J-L (1989) Effects of rearing conditions on growth and thyroid hormones during smolting of Atlantic salmon, *Salmo salar* L. Aquaculture 82: 29–38. 10.1016/0044-8486(89)90393-1.

[ref9] Boeuf G, Le Bail P-Y (1999) Does light have an influence on fish growth. Aquaculture 177: 129–152. 10.1016/S0044-8486(99)00074-5.

[ref10] Boujard T, Leatherland JF (1992) Circadian pattern of hepatosomatic index, liver glycogen and lipid content, plasma non-esterified fatty acid, glucose, T3, T4, growth hormone and cortisol concentrations in *Oncorhynchus mykiss* held under different photoperiod regimes and fed using demand-feeders. Fish Physiol Biochem 10: 111–122. 10.1007/BF00004522. 24214208

[ref11] Brown DD (1997) The role of thyroid hormone in zebrafish and axolotl development. Proc Natl Acad Sci U S A 94: 13011–13016. 10.1073/pnas.94.24.13011. 9371791 PMC24254

[ref12] Brown DRE (1952) Natural Illumination Charts. Department of the Navy, Bureau of Ships.

[ref13] Brown JA, Edwards D, Whitehead C (1989) Cortisol and thyroid hormone responses to acid stress in the brown trout, *Salmo trutta* L. J Fish Biol 35: 73–84. 10.1111/j.1095-8649.1989.tb03394.x.

[ref14] Brown SB, Adams BA, Cyr DG, Eales JG (2004) Contaminant effects on the teleost fish thyroid. Environ Toxicol Chem 23: 1680–1701. 10.1897/03-242. 15230321

[ref15] Brown SB, Eales JG, Evans RE, Hara TJ (1984) Interrenal, thyroidal, and carbohydrate responses of rainbow trout (*Salmo gairdneri*) to environmental acidification. Can J Fish Aquat Sci 41: 36–45. 10.1139/f84-004.

[ref16] Brown SB, Evans RE, Majewski HS, Sangalang GB, Klaverkarnp JF (1990) Responses of plasma electrolytes, thyroid hormones, and gill histology in Atlantic salmon *(Salmo salar)* to acid and limed river waters. Can J Fish Aquat Sci 47: 2431–2440. 10.1139/f90-271.

[ref17] Brown SB, MacLatchy DL, Hara TJ, Eales JG (1991) Effects of cortisol on aspects of 3,5,3′-triiodo-L-thyronine metabolism in rainbow trout (*Oncorhynchus mykiss*). Gen Comp Endocrinol 81: 207–216. 10.1016/0016-6480(91)90005-Q. 2019395

[ref18] Brüning A, Hölker F, Franke S, Kleiner W, Kloas W (2016) Impact of different colours of artificial light at night on melatonin rhythm and gene expression of gonadotropins in European perch. Sci Total Environ 543: 214–222. 10.1016/j.scitotenv.2015.11.023. 26584071

[ref19] Brüning A, Hölker F, Franke S, Kleiner W, Kloas W (2018a) Influence of light intensity and spectral composition of artificial light at night on melatonin rhythm and mRNA expression of gonadotropins in roach *Rutilus rutilus*. Fish Physiol Biochem 44: 1–12. 10.1007/s10695-017-0408-6. 28721487

[ref20] Brüning A, Hölker F, Franke S, Preuer T, Kloas W (2015) Spotlight on fish: light pollution affects circadian rhythms of European perch but does not cause stress. Sci Total Environ 511: 516–522. 10.1016/j.scitotenv.2014.12.094. 25577738

[ref21] Brüning A, Kloas W, Preuer T, Hölker F (2018b) Influence of artificially induced light pollution on the hormone system of two common fish species, perch and roach, in a rural habitat. Conserv Physiol 6: coy016: 10.1093/conphys/coy016. PMC5905364 29686874

[ref22] Cameron NE (1982) The photopic spectral sensitivity of a dichromatic teleost fish (*Perca fluviatilis*). Vision Res 22: 1341–1348. 10.1016/0042-6989(82)90223-1. 7157671

[ref23] Campinho MA (2019) Teleost metamorphosis: the role of thyroid hormone. Front Endocrinol 10: 383 10.3389/fendo.2019.00383. PMC6587363 31258515

[ref24] Carletta MA, Weis P, Weis JS (2002) Development of thyroid abnormalities in mummichogs, *Fundulus heteroclitus*, from a polluted site. Mar Environ Res 54: 601–604. 10.1016/S0141-1136(02)00133-2. 12408623

[ref25] Carr JA, Patiño R (2011) The hypothalamus-pituitary-thyroid axis in teleosts and amphibians: endocrine disruption and its consequences to natural populations. Gen Comp Endocrinol 170: 299–312. 10.1016/j.ygcen.2010.06.001. 20566362

[ref26] Chen R, Yuan L, Zha J, Wang Z (2017) Developmental toxicity and thyroid hormone-disrupting effects of 2,4-dichloro-6-nitrophenol in Chinese rare minnow (*Gobiocypris rarus*). Aquat Toxicol 185: 40–47. 10.1016/j.aquatox.2017.02.005. 28187359

[ref27] Cook RF, Eales JG (1987) Effects of feeding and photocycle on diel changes in plasma tyhroid hormone levels in rainbow trout, *Salmo gairdneri*. J Exp Zool 242: 161–169. 10.1002/jez.1402420207.

[ref28] Cyr DG, Bromage NR, Duston J, Eales JG (1988a) Seasonal patterns in serum levels of thyroid hormones and sex steroids in relation to photoperiod-induced changes in spawning time in rainbow trout, *Salmo gairdneri*. Gen Comp Endocrinol 69: 217–225. 10.1016/0016-6480(88)90008-1. 3366356

[ref29] Cyr DG, Eales JG (1990) Influence of short-term oestradiol treatment on plasma thyroid hormone kinetic in rainbow trout, *Salmo gairdneri*. J Fish Biol 36: 391–400. 10.1111/j.1095-8649.1990.tb05619.x.

[ref30] Cyr DG, Eales JG (1996) Interrelationships between thyroidal and reproductive endocrine systems in fish. Reviews in Fish Biology and Fisheries 6: 165–200. 10.1007/BF00182342.

[ref31] Cyr DG, McLatchy DL, Eales JG (1988b) The influence of short-term 17β-estradiol treatment on plasma T_3_ levels and *in vitro* hepatic T_4_ 5’-Monodeiodinase activity in immature rainbow trout, *Salmo gairdneri*. Gen Comp Endocrinol 69: 431–438. 10.1016/0016-6480(88)90035-4. 3360299

[ref32] Dananay KL, Benard MF (2018) Artificial light at night decreases metamorphic duration and juvenile growth in a widespread amphibian. Proc R Soc B 285: 20180367. doi: 10.1098/rspb.2018.0367. PMC6053935 30051829

[ref33] Dardente H, Hazlerigg DG, Ebling FJ (2014) Thyroid hormone and seasonal rhythmicity. Front Endocrinol 5: 19 10.3389/fendo.2014.00019. PMC3935485 24616714

[ref34] Dickhoff WW, Folmar LC, Mighell JL, Mahnken CVW (1982) Plasma thyroid hormones during smoltification of yearling and underyearling coho salmon and yearling chinook salmon and steelhead trout. Aquaculture 28: 39–48. 10.1016/0044-8486(82)90006-0.

[ref35] Eales JG, Fletcher GL (1982) Circannual cycles of thyroid hormones in plasma of winter flounder (*Pseudopleuronectes americanus* Walbaum). Can J Zool 60: 304–309. 10.1139/z82-040.

[ref36] Eales JG, Shostak S (1985) Correlations between food ration, somatic growth parameters and thyroid function in Arctic charr, *Salvelinus alpinus* L. Comp Biochem Physiol A Physiol 80A: 553–558. 10.1016/0300-9629(85)90411-6.

[ref37] Falchi F, Cinzano P, Duriscoe D, Kyba CCM, Elvidge CD, Baugh K, Portnov BA, Rybnikova NA, Furgoni R (2016) The new world atlas of artificial night sky brightness. Sci Adv 2: e1600377 10.1126/sciadv.1600377. 27386582 PMC4928945

[ref38] Falcón J, Migaud H, Muñoz-Cueto JA, Carrillo M (2010) Current knowledge on the melatonin system in teleost fish. Gen Comp Endocrinol 165: 469–482. 10.1016/j.ygcen.2009.04.026. 19409900

[ref39] Farbridge KJ, Leatherland JF (1987) Lunar cycles of coho salmon, *Oncorhynchus kisutch* - II. Scale amino acid uptake, nucleic acids, metabolic reserves and plasma thyroid hormones. J Exp Biol 129: 179–189. 2438366 10.1242/jeb.129.1.179

[ref40] Fort DJ, Rogers RL, Morgan LA, Miller MF, Clark PA, White JA, Paul RR, Stover EL (2000) Preliminary validation of a short-term morphological assay to evaluate adverse effects of amphibian metamorphosis and thyroid function using *Xenopus laevis*. J Appl Toxicol 20: 419–425. https://doi.org/10.1002/1099-1263(200009/10)20:5419::AID-JAT7083.0.CO;2-A. 11139173 10.1002/1099-1263(200009/10)20:5<419::AID-JAT708>3.0.CO;2-A

[ref41] Franke S, Brüning A, Hölker F, Kloas W (2013) Study of biological action of light on fish. J Light Vis Environ 37: 194–204. 10.2150/jlve.IEIJ130000518.

[ref42] Gaston KJ, Duffy JP, Gaston S, Bennie J, Davies TW (2014) Human alteration of natural light cycles: causes and ecological consequences. Oecologia 176: 917–931. doi: 10.1007/s00442-014-3088-2. 25239105 PMC4226844

[ref43] Gaston KJ, Visser ME, Hölker F (2015) The biological impacts of artificial light at night: the research challenge. Philos Trans R Soc Lond B Biol Sci 370: 20140133 10.1098/rstb.2014.0133. 25780244 PMC4375372

[ref44] Gomez JM, Boujard T, Boeuf G, Solari A, Le Bail P-Y (1997) Individual diurnal plasma profiles of thyroid hormones in rainbow trout *(Oncorhynchus mykiss)* in relation to cortisol, growth hormone, and growth rate. Gen Comp Endocrinol 107: 74–83. 10.1006/gcen.1997.6897. 9208307

[ref45] Grau EG (1988) Environmental influences on thyroid function of teleost fish. Am Zool 329–335. 10.1093/icb/28.2.329.

[ref46] Grau EG, Dickhoff WW, Nishioka RS, Bern HA, Folmar LC (1981) Lunar phasing of the thyroxine surge preparatory to seaward migration salmonid fish. Science 211: 607–609. 10.1126/science.7455703. 7455703

[ref47] Grubisic M et al. (2019) Light pollution, circadian photoreception, and melatonin in vertebrates. Sustainability 11: 6400 10.3390/su11226400.

[ref48] Grubisic M, Singer G, Bruno MC, van Grunsven RHA, Manfrin A, Monaghan MT, Hölker F (2017) Artificial light at night decreases biomass and alters community composition of benthic primary producers in a sub-alpine stream. Limnol Oceanogr 62: 2799–2810. 10.1002/lno.10607.

[ref49] Hänel A et al. (2018) Measuring night sky brightness: methods and challenges. J Quant Spectrosc Radiat Transf 205: 278–290. 10.1016/j.jqsrt.2017.09.008.

[ref50] Hölker F, Jechow A, Schroer S, Gessner MO (2018) Nächtliches Licht und Lichtverschmutzung in und um Gewässer. Handbuch Angewandte Limnologie 34: 1–26. 10.1002/9783527678488.hbla2018003.

[ref51] Hölker F, Wolter C, Perkin EK, Tockner K (2010) Light pollution as a biodiversity threat. Trends Ecol Evol 25: 681–682. 10.1016/j.tree.2010.09.007. 21035893

[ref52] Hölker F, Wurzbacher C, Weißenborn C, Monaghan MT, Holzhauer SI, Premke K (2015) Microbial diversity and community respiration in freshwater sediments influenced by artificial light at night. Philos Trans R Soc Lond B Biol Sci 370: 20140130 10.1098/rstb.2014.0130. 25780242 PMC4375370

[ref53] Hoseini SM, Tort L, Abolhasani MH, Rajabiesterabadi H (2016) Physiological, ionoregulatory, metabolic and immune responses of Persian sturgeon, *Acipenser persicus* (Borodin, 1897) to stress. Aquacult Res 47: 3729–3739. 10.1111/are.12822.

[ref54] Ikeno T, Weil ZM, Nelson RJ (2014) Dim light at night disrupts the short-day response in Siberian hamsters. Gen Comp Endocrinol 197: 56–64. 10.1016/j.ygcen.2013.12.005. 24362257

[ref55] Inui Y, Miwa S (1985) Thyroid hormone induces metamorphosis of flounder larvae. Gen Comp Endocrinol 60: 450–454. 10.1016/0016-6480(85)90080-2. 3935513

[ref56] Jung SJ, Kim NN, Choi YJ, Choi JY, Choi YU, Heo YS, Choi CY (2016) Effects of melatonin and green-wavelength LED light on the physiological stress and immunity of goldfish, *Carassius auratus*, exposed to high water temperature. Fish Physiol Biochem 42: 1335–1346. 10.1007/s10695-016-0221-7. 27012684

[ref57] Kloas W (2002) Amphibians as a model for the study of endocrine disruptors. Int Rev Cytol 216: 1–57. 10.1016/S0074-7696(02)16002-5. 12049206

[ref58] Kloas W et al. (2009) Endocrine disruption in aquatic vertebrates. Ann N Y Acad Sci 1163: 187–200. 10.1111/j.1749-6632.2009.04453.x. 19456339

[ref59] Kummu M, de Moel H, Ward PJ, Varis O (2011) How close do we live to water? A global analysis of population distance to freshwater bodies. PLoS One 6: e20578 10.1371/journal.pone.0020578. 21687675 PMC3110782

[ref60] Kupprat F, Hölker F, Kloas W (2020) Can skyglow reduce nocturnal melatonin concentrations in Eurasian perch? Environ Pollut 262: 114324 10.1016/j.envpol.2020.114324. 32179225

[ref61] Kyba CCM, Hänel A, Hölker F (2014) Redefining efficiency for outdoor lighting. Energ Environ Sci 7: 1806–1809. 10.1039/c4ee00566j.

[ref62] Laidley CW, Leatherland JF (1988) Circadian studies of plasma cortisol, thyroid hormone, protein, glucose and ion concentration, liver glycogen concentration, and liver and spleen weight in rainbow trout, *Salmo gairdneri* Richardson. Comp Biochem Physiol 89: 495–502. 10.1016/0300-9629(88)91063-8. 2896578

[ref63] Laudet V (2011) The origins and evolution of vertebrate metamorphosis. Curr Biol 21: R726–R737. 10.1016/j.cub.2011.07.030. 21959163

[ref64] Leatherland JF (1985) Effect of 17β-estradiol and methyl testosterone on the activity of the thyroid gland in rainbow trout, *Salmo* gairdneri Richardson. Gen Comp Endocrinol 60: 343–352. 10.1016/0016-6480(85)90067-X. 4076757

[ref65] Leatherland JF (1994) Reflections on the Thyroidology of Fishes: From Molecules to Humankind. Institute of Ichthyology, University of Guelph, Guelph, Ontario, Canada.

[ref66] Leatherland JF, Sonstegard RA (1978) Lowering of serum thyroxine and triiodothyronine levels in Coho salmon, *Oncorhynchus kisutch*, by dietary mirex and PCBs. J Fish Res Board Can 35: 1285–1289. 10.1139/f78-202.

[ref67] Leiner KA, MacKenzie DS (2001) The effects of photoperiod on growth rate and circulating thyroid hormone levels in the red rum, *Sciaenops ocellatus*: evidence for a free-running circadian rhythm of T4 secretion. Comp Biochem Physiol A Mol Integr Physiol 130: 141–149. 10.1016/S1095-6433(01)00373-7. 11672690

[ref68] Lenth R (2019). Emmeans: estimated marginal means, aka least-squares means. R package version 1.3.3.

[ref69] Lorgen M et al. (2015) Functional divergence of type 2 deiodinase paralogs in the Atlantic salmon. Curr Biol 25: 936–941. 10.1016/j.cub.2015.01.074. 25802152

[ref70] Manfrin A, Singer G, Larsen S, Weiß N, van Grunsven RHA, Weiß N-S, Wohlfahrt S, Monaghan MT, Hölker F (2017) Artificial light at night affects organism flux across ecosystem boundaries and drives community structure in the recipient ecosystem. Front Environ Sci 5: 61 10.3389/fenvs.2017.00061.

[ref71] McCormick SD, Saunders RL (1990) Influence of ration level and salinity on circulating thyroid hormones in juvenile Atlantic salmon (*Salmo salar*). Gen Comp Endocrinol 78: 224–230. 10.1016/0016-6480(90)90009-B. 2354765

[ref72] McCormick SD, Saunders RL, Henderson EB, Harmon PR (1987) Phototoperiod control of parr–smolt transformation in Atlantic salmon (*Salmo salar*): changes in salinity tolerance, gill Na^+^, K^+^-ATPase activity, and plasma thyroid hormones. Can J Fish Aquat Sci 44: 1462–1468. 10.1139/f87-175.

[ref73] Miwa S, Inui Y (1987) Effects of various doses of thyroxine and triiodothyronine on the metamorphosis of flounder (*Paralichthys olivaceus*). Gen Comp Endocrinol 67: 356–363. 10.1016/0016-6480(87)90190-0. 3666411

[ref74] Mukhi S, Patiño R (2007) Effects of prolonged exposure to perchlorate on thyroid and reproductive function in zebrafish. Toxicol Sci 96: 246–254. 10.1093/toxsci/kfm001. 17205975

[ref75] Nakane Y et al. (2013) The saccus vasculosus of fish is a sensor of seasonal changes in day length. Nat Commun 4: 2108 10.1038/ncomms3108. 23820554

[ref76] Nakane Y, Yoshimura T (2014) Universality and diversity in the signal transduction pathway that regulates seasonal reproduction in vertebrates. Front Neurosci 8: 115 10.3389/fnins.2014.00115. 24959116 PMC4033074

[ref77] Nakao N et al. (2008) Thyrotrophin in the pars tuberalis triggers photoperiodic response. Nature 452: 317–322. 10.1038/nature06738. 18354476

[ref78] Noyes PD, Lema SC, Roberts SC, Cooper EM, Stapleton HM (2014) Rapid method for the measurement of circulating thyroid hormones in low volumes of teleost fish plasma by LC-ESI/MS/MS. Anal Bioanal Chem 406: 715–726. 10.1007/s00216-013-7528-3. 24343452 PMC4056446

[ref79] O'Brien CS, Bourdo R, Bradshaw WE, Holzapfel CM, Cresko WA (2012) Conservation of the photoperiodic neuroendocrine axis among vertebrates: evidence from the teleost fish, Gasterosteus aculeatus. Gen Comp Endocrinol 178: 19–27. 10.1016/j.ygcen.2012.03.010. 22504272 PMC3389224

[ref80] OECD (2009) Test No. 231: Amphibian metamorphosis assay In OECD Guidelines for the Testing of Chemicals. OECD Publishing, Paris 10.1787/9789264076242-en.

[ref81] Okumoto N, Ikuta K, Aida K, Hanyu I, Hirano T (1989) Effects of photoperiod on smolting and hormonal secretion in masu salmon, *Oncorhynchus masou*. Aquaculture 82: 63–76. 10.1016/0044-8486(89)90396-7.

[ref82] Opitz R, Braunbeck T, Bogi C, Pickford DB, Nentwig G, Oehlmann J, Tooi O, Lutz I, Kloas W (2005) Description and initial evaluation of a *Xenopus* metamorphosis assay for detection of thyroid system-disrupting activities of environmental compounds. Environ Toxicol Chem 24: 653–664. 10.1897/04-214R.1. 15779766

[ref83] Orozco A, Valverde-R C (2005) Thyroid hormone deiodination in fish. Thyroid 15: 799–813. 10.1089/thy.2005.15.799. 16131323

[ref84] Osborn RH, Simpson TH (1978) Seasonal changes in thyroidal status in the plaice *Pleuronectes platessa* L. J Fish Biol 12: 519–526. 10.1111/j.1095-8649.1978.tb04197.x.

[ref85] Osborn RH, Simpson TH, Youngson AF (1978) Seasonal and diurnal rhythms of thyroidal status in the rainbow trout, *Salmo gairdneri* Richardson. J Fish Biol 12: 531–540. 10.1111/j.1095-8649.1978.tb04199.x.

[ref86] Ouyang JQ, Davies S, Dominoni D (2018) Hormonally mediated effects of artificial light at night on behavior and fitness: linking endocrine mechanisms with function. J Exp Biol 221: jeb156893 10.1242/jeb.156893. PMC5897701 29545373

[ref87] Özeren SC, Kankılıç GB, Erkmen B, Polat H, Pehlivan E (2019) Effect of seasonal water temperature variation on the blood serums thyroid hormone levels of juvenile chub fishes (*Squalius cappadocicus*).Biol Rhythm Res 1–6. 10.1080/09291016.2019.1566987.

[ref88] Peter VS, Joshua EK, Wendelaar Bonga SE, Peter MC (2007) Metabolic and thyroidal response in air-breathing perch *(Anabas testudineus)* to water-borne kerosene. Gen Comp Endocrinol 152: 198–205. 10.1016/j.ygcen.2007.05.015. 17574248

[ref89] Power DM, Llewellyn L, Faustino M, Nowell MA, Björnsson BT, Einarsdottir IE, Canario AVM, Sweeney GE (2001) Thyroid hormones in growth and development of fish. Comp Biochem Physiol C-Toxicol Pharmacol 130: 447–459. 10.1016/S1532-0456 (01)00271-X. 11738632

[ref90] Reddy PK, Leatherland JF (2003) Influences of photoperiod and alternate days of feeding on plasma growth hormone and thyroid hormone levels in juvenile rainbow trout. J Fish Biol 63: 197–212. 10.1046/j.1095-8649.2003.00144.x.

[ref91] Reid AJ et al. (2018) Emerging threats and persistent conservation challenges for freshwater biodiversity. Biol Rev Camb Philos Soc 94: 849–873. 10.1111/brv.12480. 30467930

[ref92] Riley WD, Bendall B, Ives MJ, Edmonds NJ, Maxwell DL (2012) Street lighting disrupts the diel migratory pattern of wild Atlantic salmon, *Salmo salar* L., smolts leaving their natal stream. *Aquaculture* 330-333: 74-81. Doi: 10.1016/j.aquaculture.2011.12.009

[ref93] Riley WD, Davison PI, Maxwell DL, Bendall B (2013) Street lighting delays and disrupts the dispersal of Atlantic salmon (*Salmo salar*) fry. Biol Conserv 158: 140–146. 10.1016/j.biocon.2012.09.022.

[ref94] Schreiber AM (2006) Asymmetric craniofacial remodeling and lateralized behavior in larval flatfish. J Exp Biol 209: 610–621. 10.1242/jeb.02056. 16449556

[ref95] Schroer S, Huggins BJ, Azam C, Hölker F (2020) Working with inadequate tools: legislative shortcomings in protection against ecological effects of artificial light at night. Sustainability 12: 2551 10.3390/su12062551.

[ref96] Touzot M, Lengagne T, Secondi J, Desouhant E, Théry M, Dumet A, Duchamp C, Mondy N (2020) Artificial light at night alters the sexual behaviour and fertilisation success of the common toad. Environ Pollut 259: 113883 10.1016/j.envpol.2019.113883. 31931411

[ref97a] Vivas Muñoz JC, Bierbach D, Knopf K (2019) Eye fluke *(Tylodelphys clavata)* infection impairs visual ability and hampers foraging success in European perch. Parasitol Res 118: 2531–2541. 10.1007/s00436-019-06389-5. 31286263

[ref97] Vriend J, Reiter RJ, Anderson GR (1979) Effects of the pineal and melatonin on thyroid activity of male golden hamsters. Gen Comp Endocrinol 38: 189–195. 10.1016/0016-6480(79)90206-5. 488673

[ref98] Walpita CN, Crawford AD, Janssens ED, Van der Geyten S, Darras VM (2009) Type 2 iodothyronine deiodinase is essential for thyroid hormone-dependent embryonic development and pigmentation in zebrafish. Endocrinology 150: 530–539. 10.1210/en.2008-0457. 18801906

[ref99] Walpita CN, Van der Geyten S, Rurangwa E, Darras VM (2007) The effect of 3,5,3′-triiodothyronine supplementation on zebrafish *(Danio rerio)* embryonic development and expression of iodothyronine deiodinases and thyroid hormone receptors. Gen Comp Endocrinol 152: 206–214. 10.1016/j.ygcen.2007.02.020. 17418841

[ref100] Wang D, Stapleton HM (2010) Analysis of thyroid hormones in serum by liquid chromatography-tandem mass spectrometry. Anal Bioanal Chem 397: 1831–1839. 10.1007/s00216-010-3705-9. 20437035 PMC3082288

[ref101] Weber GM, Okimoto DK, Richman NH III, Grau EG (1992) Patterns of thyroxine and triiodothyronine in serum and follicle-bound oocytes of the tilapia, *Oreochromis mossambicus*, during oogenesis. Gen Comp Endocrinol 85: 392–404. 10.1016/0016-6480(92)90084-w. 1577243

[ref102] Wittkowski W, Bergmann M, Hoffmann K, Pera F (1988) Photoperiod-dependent changes in TSH-like immunoreactivity of cells in the hypophysial pars tuberalis of the Djungarian hamster, *Phodopus sungorus*. Cell Tissue Res 251: 183–187. 10.1007/BF00215463. 3342436

[ref103] Wood S, Loudon A (2014) Clocks for all seasons: unwinding the roles and mechanisms of circadian and interval timers in the hypothalamus and pituitary. J Endocrinol 222: R39–R59 10.1530/JOE-14-0141. 24891434 PMC4104039

[ref104a] Wright ML (2002) Melatonin, diel rhythms, and metamorphosis in anuran amphibians. Gen Comp Endocrinol 126: 251–254. 10.1016/S0016-6480(02)00012-6. 12093111

[ref104] Yoshimura T (2010) Neuroendocrine mechanism of seasonal reproduction in birds and mammals. Anim Sci J 81: 403–410. 10.1111/j.1740-0929.2010.00777.x. 20662808

[ref105] Zhao X, Ren X, Ren B, Luo Z, Zhu R (2016) Life-cycle exposure to BDE-47 results in thyroid endocrine disruption to adults and offsprings of zebrafish (*Danio rerio*). Environ Toxicol Pharmacol 48: 157–167. 10.1016/j.etap.2016.10.004. 27780123

[ref106] Zhou T, John-Alder HB, Weis JS, Weis P (2000) Endocrine disruption: thyroid dysfunction in mummichogs (*Fundulus heteroclitus*) from a polluted habitat. Mar Environ Res 50: 393–397. 10.1016/S0141-1136(00)00042-8. 11460725

[ref107] Zuur AF, Ieno EN, Walker NJ, Saveliev AA, Smith GM (2009) Mixed Effects Models and Extensions in Ecology with R. Springer Science+Business Media, LLC, New York, USA.

