# Supplementary material for: Misbalance of thyroid hormones after two weeks of exposure to artificial light at night in Eurasian perch Perca fluviatilis
Source: Conserv Physiol. 2021 Jan 7;9(1):coaa124. doi: 10.1093/conphys/coaa124 (PMC7905158; doi:10.1093/conphys/coaa124)
Supplement: Kupprat_et_al_ConsPhys_Supplementary_material_re-revised_coaa124 [file kupprat_et_al_consphys_supplementary_material_re-revised_coaa124.docx]

**Supplementary material to “Misbalance of thyroid hormones after two weeks of exposure to artificial light at night in Eurasian perch *Perca fluviatilis”***

**Abbreviations for Tables S1 to S3**

ALAN – Artificial light at night

LMM – Linear mixed model

R²_m_ – marginal R²

R²_c_ – conditional R²

SE – standard error

df – degrees of freedom

LLR – log-likelihood ratio

f – female

m – male

nd – not differentiated (premature fish)

**Table S1**

**LMM specifications and post-hoc results (Tukey’s correction) for significant fixed effects in plasma triiodothyronine (T3) of *Perca fluviatilis* exposed to different nocturnal light intensities in two different experiments.**

| **log(T3) high ALAN experiment (R²_m_ = 0.2894, R²_c_ = 0.3548)** | | | | | |
| --- | --- | --- | --- | --- | --- |
| **Fixed effects** | **Estimate ± SE** | **df** | **t-value** | **LLR** | **p-value** |
| **Intercept** ‡‡ | 1.231 ± 0.171 | 83 | 7.18 |  |  |
| **ALAN 1 lux** | -0.097 ± 0.085 | 15 | -1.14 | 11.94 | 0.0076 |
| **ALAN 10 lux** | 0.075 ± 0.086 | 15 | -0.87 |  |  |
| **ALAN 100 lux** | -0.309 ± 0.085 | 15 | -3.63 |  |  |
| Sex m | 0.377 ± 0.079 | 83 | 4.74 | 24.34 | <0.0001 |
| Sex nd | 0.062 ± 0.074 | 83 | 0.83 |  |  |
| Body mass | 0.007 ± 0.002 | 83 | 3.65 | 12.47 | 0.0004 |
| **Random effect** | **Estimate** |  |  | **LLR** | **p-value** |
| Runs | σ² = 0.08² |  |  |  |  |
| Aquaria nested in runs | σ² = 0.05² |  |  | 2.08 | 0.3536 |
| Residual | σ² = 0.29² |  |  |  |  |
| **Post-hoc treatment effects** | **Estimate ± SE** | **df** | **t-ratio** |  | **p-value** |
| 0 lx – 1 lx | 0.097 ± 0.086 | 15 | 1.14 |  | 0.6722 |
| 0 lx – 10 lx | 0.075 ± 0.086 | 15 | 0.87 |  | 0.8202 |
| 0 lx – 100 lx | 0.309 ± 0.085 | 15 | 3.63 |  | 0.0118 |
| 1 lx – 10 lx | -0.022 ± 0.085 | 15 | -0.26 |  | 0.9934 |
| 1 lx – 100 lx | 0.212 ± 0.085 | 15 | 2.50 |  | 0.0999 |
| 10 lx – 100 lx | 0.234 ± 0.085 | 15 | 2.74 |  | 0.0652 |
| **Post-hoc sex effects** | **Estimate ± SE** | **df** | **t-ratio** |  | **p-value** |
| f – m | -0.377 ± 0.080 | 83 | -4.74 |  | <0.0001 |
| f – nd | -0.062 ± 0.074 | 83 | 0.84 |  | 0.6825 |
| m – nd | 0.315 ± 0.073 | 83 | 4.33 |  | 0.0001 |
| **log(T3) low ALAN experiment (R²_m_ = 0.1546, R²_c_ = 0.4138)** | | | | | |
| **Fixed effects** | **Estimate ± SE** | **df** | **t-value** | **LLR** | **p-value** |
| **Intercept** † | -0.608 ± 0.260 | 115 | -2.34 |  |  |
| **ALAN 0.01 lx** | -0.025 ± 0.083 | 19 | -0.30 | 1.05 | 0.7886 |
| **ALAN 0.1 lx** | -0.020 ± 0.082 | 19 | -0.24 |  |  |
| **ALAN 1 lx** | -0.080 ± 0.083 | 19 | -0.96 |  |  |
| Body mass | 0.044 ± 0.089 | 115 | 4.76 | 22.07 | <0.0001 |
| **Random effect** | **Estimate** |  |  | **LLR** | **p-value** |
| Runs | σ² = 0.23² |  |  |  |  |
| Aquaria nested in runs | σ² = 9.03e-06² |  |  | 15.52 | 0.0004 |
| Residual | σ² = 0.34² |  |  |  |  |

‡ Intercet taken at 0 lx for females

† Intercept taken at 0 lx

**Table S2**

**LMM specifications for plasma thyroxine (T4) of *Perca fluviatilis* exposed to different nocturnal light intensities in two different experiments.**

| **log(T4) high ALAN experiment (R²_m_ = 0.0246, R²_c_ = 0.1648)** | | | | | |
| --- | --- | --- | --- | --- | --- |
| **Fixed effects** | **Estimate ± SE** | **df** | **t-value** | **LLR** | **p-value** |
| **Intercept** † | 0.962 ± 0.108 | 84 | 8.93 |  |  |
| **ALAN 1 lux** | 0.063 ± 0.152 | 15 | 0.41 | 1.96 | 0.5809 |
| **ALAN 10 lux** | 0.156 ± 0.152 | 15 | 1.03 |  |  |
| **ALAN 100 lux** | 0.164 ± 0.152 | 15 | 1.08 |  |  |
| **Random effect** | **Estimate** |  |  | **LLR** | **p-value** |
| Runs | σ² = 2.4e-05² |  |  |  |  |
| Aquaria nested in runs | σ² = 0.16² |  |  | 2.83 | 0.2429 |
| Residual | σ² = 0.40² |  |  |  |  |
| **log(T4) low ALAN experiment (R²_m_ = 0.1633, R²_c_ = 0.1873)** | | | | | |
| **Fixed effects** | **Estimate ± SE** | **df** | **t-value** | **LLR** | **p-value** |
| **Intercept** † | 1.388 ± 0.471 | 73 | 2.95 |  |  |
| **ALAN 0.01 lx** | -2.411 ± 0.804 | 19 | -3.00 |  |  |
| **ALAN 0.1 lx** | -1.986 ± 0.743 | 19 | -2.67 |  |  |
| **ALAN 1 lx** | -0.041 ± 0.782 | 19 | -0.05 |  |  |
| **Body mass** | -0.044 ± 0.022 | 73 | -2.00 |  |  |
| **ALAN 0.01 lx : Body mass** | 0.117 ± 0.039 | 73 | 2.97 | 14.70 | 0.0021 |
| **ALAN 0.1 lx : Body mass** | 0.101 ± 0.036 | 73 | 2.80 |  |  |
| **ALAN 1 lx : Body mass** | -0.008 ± 0.038 | 73 | -0.22 |  |  |
| **Random effect** | **Estimate** |  |  | **LLR** | **p-value** |
| Runs | σ² = 1.64e-05² |  |  |  |  |
| Aquaria nested in runs | σ² = 0.08² |  |  | 0.10 | 0.9498 |
| Residual | σ² = 0.49² |  |  |  |  |
| **Post-hoc treatment effects** | **Estimate ± SE** | **df** | **t-ratio** |  | **p-value** |
| 0 lx – 0.01 lx | -0.117 ± 0.039 | 73 | -2.97 |  | 0.0203 |
| 0 lx – 0.1 lx | -0.101 ± 0.036 | 73 | -2.80 |  | 0.0326 |
| 0 lx – 1 lx | 0.008 ± 0.038 | 73 | 0.22 |  | 0.9960 |
| 0.01 lx – 0.1 lx | 0.016 ± 0.043 | 73 | 0.36 |  | 0.9835 |
| 0.01 lx – 1 lx | 0.125 ± 0.045 | 73 | 2.81 |  | 0.0315 |
| 0.1 lx – 1 lx | 0.110 ± 0.042 | 73 | 2.62 |  | 0.0513 |

† Intercept taken at 0 lx

**Table S3**

**LMM specifications for the ratio of triiodothyronine (T3) and thyroxine (T4) in the plasma of *Perca fluviatilis* exposed to different nocturnal light intensities in two different experiments.**

| **log(T3/T4) high ALAN experiment (R²_m_ = 0.0845, R²_c_ = 0.1957)** | | | | | |
| --- | --- | --- | --- | --- | --- |
| **Fixed effects** | **Estimate ± SE** | **df** | **t-value** | **LLR** | **p-value** |
| **Intercept** † | 0.884 ± 0.141 | 84 | 6.26 |  |  |
| **ALAN 1 lux** | -0.124 ± 0.200 | 15 | -0.62 | 6.58 | 0.0867 |
| **ALAN 10 lux** | -0.254 ± 0.200 | 15 | -1.28 |  |  |
| **ALAN 100 lux** | -0.478 ± 0.200 | 15 | -2.39 |  |  |
| **Random effect** | **Estimate** |  |  | **LLR** | **p-value** |
| Runs | σ² = 1.82e-05² |  |  |  |  |
| Aquaria nested in runs | σ² = 0.20² |  |  | 2.16 | 0.3393 |
| Residual | σ² = 0.55² |  |  |  |  |
| **log(T3/T4) low ALAN experiment (R²_m_ = 0.1306, R²_c_ = 0.3217)** | | | | | |
| **Fixed effects** | **Estimate ± SE** | **df** | **t-value** | **LLR** | **p-value** |
| **Intercept** † | -1.348 ± 0.612 | 73 | -2.20 |  |  |
| **ALAN 0.01 lx** | 1.815 ± 0.924 | 19 | 1.96 |  |  |
| **ALAN 0.1 lx** | 0.955 ± 0.855 | 19 | 1.12 |  |  |
| **ALAN 1 lx** | -0.978 ± 0.898 | 19 | -1.09 |  |  |
| **Body mass** | 0.061 ± 0.027 | 73 | 2.26 |  |  |
| **ALAN 0.01 lx : Body mass** | -0.090 ± 0.045 | 73 | -2.00 | 9.33 | 0.0252 |
| **ALAN 0.1 lx : Body mass** | -0.053 ± 0.042 | 73 | -1.27 |  |  |
| **ALAN 1 lx : Body mass** | 0.051 ± 0.043 | 73 | 1.18 |  |  |
| **Random effect** | **Estimate** |  |  | **LLR** | **p-value** |
| Runs | σ² = 0.28² |  |  |  |  |
| Aquaria nested in runs | σ² = 0.10² |  |  | 5.19 | 0.0747 |
| Residual | σ² = 0.56² |  |  |  |  |
| **Post-hoc treatment effects** | **Estimate ± SE** | **df** | **t-ratio** |  | **p-value** |
| 0 lx – 0.01 lx | 0.091 ± 0.045 | 73 | 2.00 |  | 0.1968 |
| 0 lx – 0.1 lx | 0.053 ± 0.042 | 73 | 1.27 |  | 0.5867 |
| 0 lx – 1 lx | -0.051 ± 0.043 | 73 | -1.18 |  | 0.6423 |
| 0.01 lx – 0.1 lx | -0.038 ± 0.050 | 73 | -0.76 |  | 0.8741 |
| 0.01 lx – 1 lx | -0.142 ± 0.051 | 73 | -2.76 |  | 0.0359 |
| 0.1 lx – 1 lx | -0.104 ± 0.048 | 73 | -2.16 |  | 0.1449 |

† Intercept taken at 0 lx

**Table S4**

**Sample numbers per ALAN treatment, per sex, and per hormone (T3 or T4) of analyzed plasma samples of *Perca fluviatilis* exposed to different nocturnal light intensities in two different experiments.** Numbers in brackets are samples that were extracted and measured but with values below the limit of quantification.

| **high ALAN experiment** | | | | | | | | | | |
| --- | --- | --- | --- | --- | --- | --- | --- | --- | --- | --- |
|  | **0 lx** | | **1 lx** | | **10 lx** | | **100 lx** | | **all ALAN treatments** | |
|  | T3 | T4 | T3 | T4 | T3 | T4 | T3 | T4 | **T3** | **T4** |
| females | 9 | 9 | 8 | 8 | 8 | 8 | 9 | 9 | **34** | **34** |
| males | 11 | 11 | 10 | 10 | 8 | 8 | 11 | 10  (+1) | **40** | **39**  **(+1)** |
| not differentiated | 6 | 6 | 9 | 8  (+1) | 10 | 10 | 7 | 7 | **32** | **31**  **(+1)** |
| **all sexes** | **26** | **26** | **27** | **26**  **(+1)** | **26** | **26** | **27** | **26**  **(+1)** | **106** | **104**  **(+2)** |
| **low ALAN experiment** | | | | | | | | | | |
|  | **0 lx** | | **0.01 lx** | | **0.1 lx** | | **1 lx** | | **all ALAN treatments** | |
|  | T3 | T4 | T3 | T4 | T3 | T4 | T3 | T4 | **T3** | **T4** |
| females | 3 | 2  (+1) | 1 | 0  (+1) | 1 | 0  (+1) | 3 | 3 | **8** | **5** |
| males | 17 | 12  (+5) | 19  (+1) | 16  (+4) | 15 | 13  (+2) | 15 | 11  (+4) | **66**  **(+1)** | **52**  **(+15)** |
| not differentiated | 13  (+1) | 11  (+3) | 15 | 9  (+6) | 20 | 15  (+5) | 15 | 9  (+6) | **63 (+1)** | **44**  **(+20)** |
| not available | 1 | 1 | 0 | 0 | 0 | 0 | 2 | 0  (+2) | **3** | **1**  **(+2)** |
| **all sexes** | **34**  **(+1)** | **26**  **(+9)** | **35**  **(+1)** | **25**  **(+11)** | **36** | **28**  **(+8)** | **35** | **23**  **(+12)** | **140**  **(+2)** | **102**  **(+38)** |

**
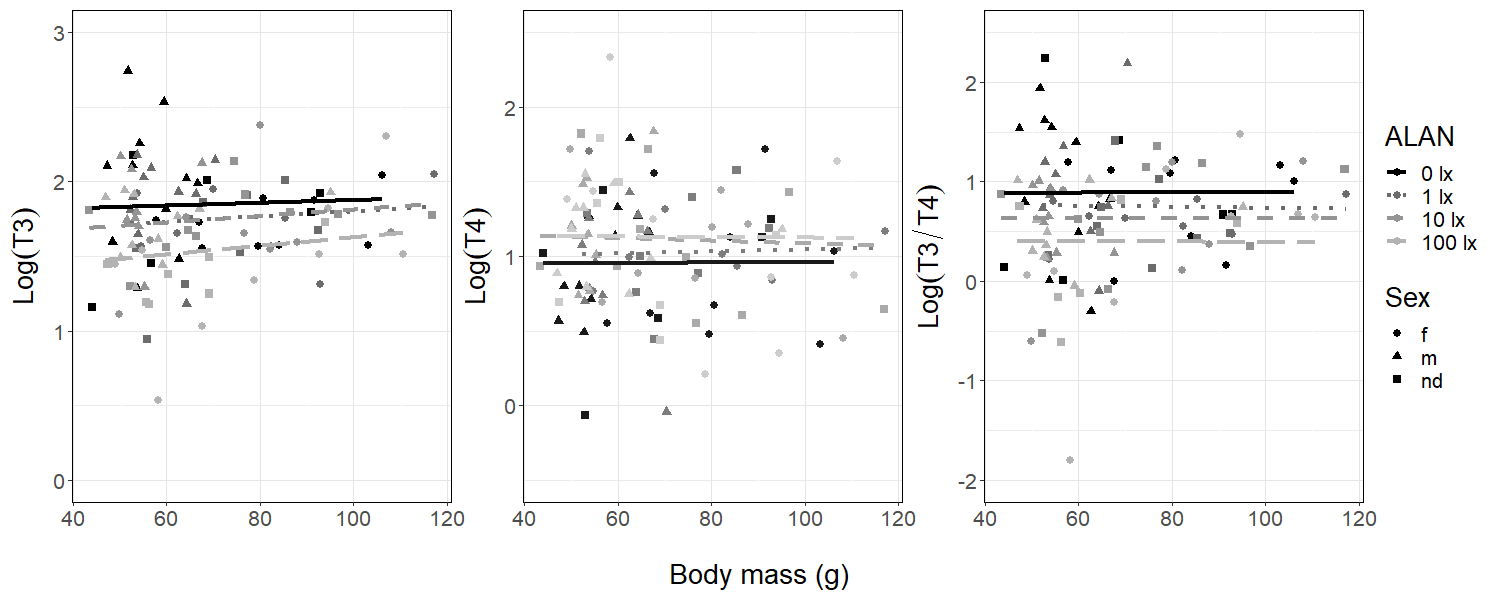
**

**Figure S1**

**Logarithmized T3 (ng/mL), T4 (ng/mL), or ratio of T3/T4 against body mass (g) of *Perca fluviatilis* exposed to different nocturnal light intensities (different shades of grey) for different sexes (shapes) (“high ALAN experiment”).** Lines depict the predictions of LMM analyses for each ALAN treatment (see tables S1-S3 above). Artificial light at night (ALAN) and sex, as well as body mass significantly explained variance only of log(T3) (ALAN effects: LLR = 11.94, p = 0.008; Sex effect: LLR = 24.34, p < 0.0001; Body mass effect: LLR = 12.47, p = 0.0004). ALAN did not significantly explain variance of T4 or T3/T4 (T4: LLR = 1.96, p = 0.58; T3/T4: LLR = 6.38, p = 0.09).

**
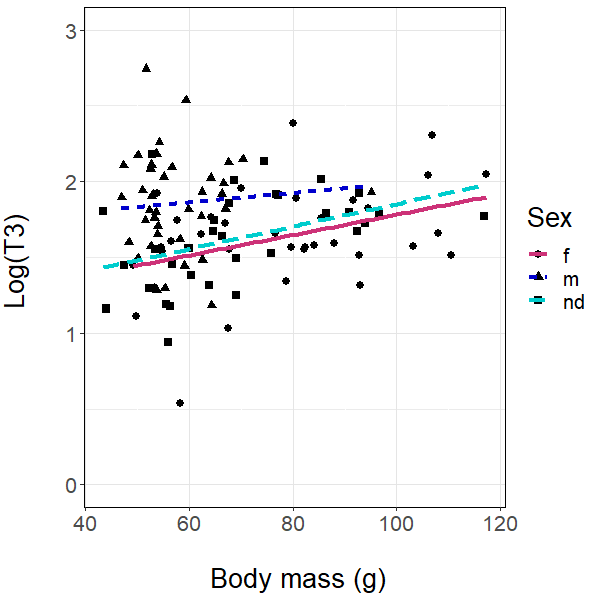
**

**Figure S2**

**Logarithmized T3 (ng/mL), T4 (ng/mL), or ratio of T3/T4 against body mass (g) of *Perca fluviatilis* exposed to different nocturnal light intensities for different sexes (shapes) (“high ALAN experiment”).** Lines depict the predictions of LMM analyses for females (f; solid), males (m; dotted) and not differentiated (nd; dashed). Sex significantly explained T3 variance (fixed effect of sex: LLR = 24.34, p < 0.0001).

**
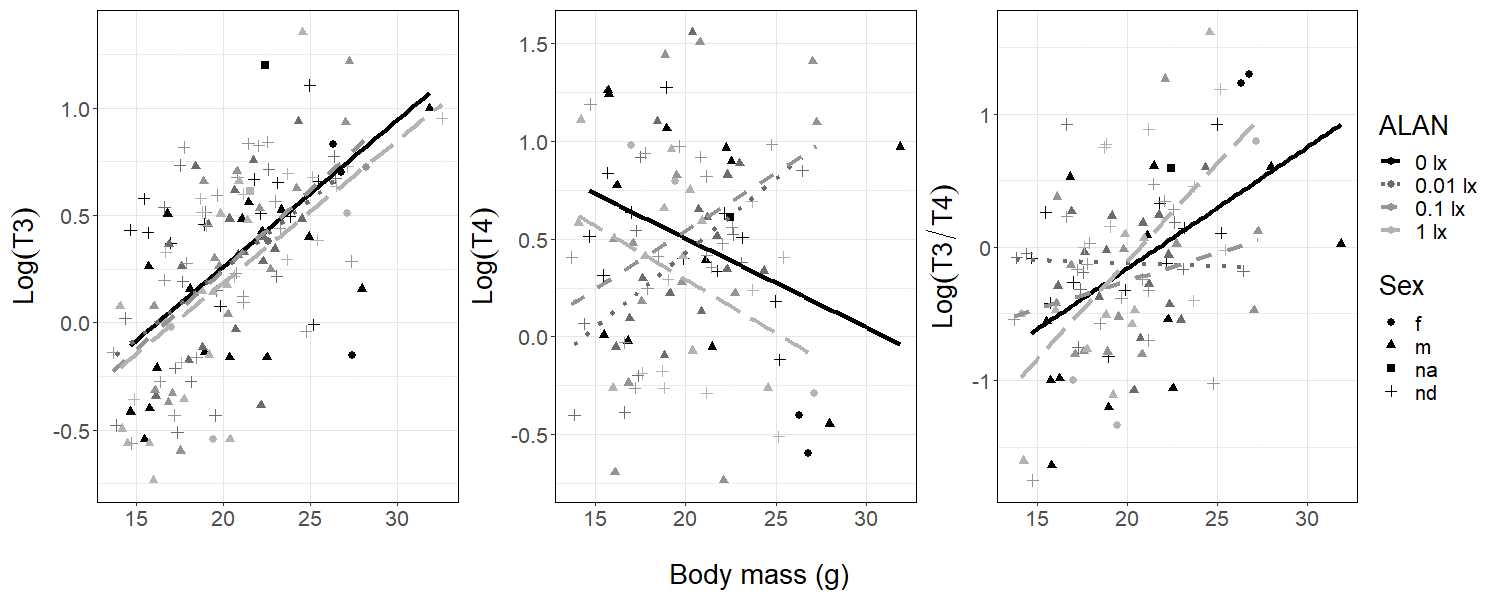
**

**Figure S3**

**Logarithmized triiodothyronine (ng/mL) (Log(T3)), thyroxine (ng/mL) (Log(T4)), or ratio of T3/T4 (Log(T3/T4)) against body mass (g) of *Perca fluviatilis* exposed to different nocturnal light intensities (different shades of grey) for different sexes (shapes) (“low ALAN experiment”).** Lines depict the predictions of LMM analyses for each ALAN treatment (see tables S1-S3 above). Artificial light at night (ALAN) did not significantly explain variance of log(T3) (ALAN effect: LLR = 1.05, p = 0.79). Body mass significantly explained variance of log(T3) (Body mass effect: LLR = 22.07, p < 0.0001). An interaction of ALAN and body mass significantly explained variance of T4 or T3/T4 (T4: LLR = 14.70, p = 0.002; T3/T4: LLR = 9.33, p = 0.03).
